# Next-Generation Sequencing of Chinese Children with Congenital Hearing Loss Reveals Rare and Novel Variants in Known and Candidate Genes

**DOI:** 10.3390/biomedicines12122657

**Published:** 2024-11-21

**Authors:** Yuan Jin, Xiaozhou Liu, Qiong Zhang, Ying Xiong, Yao Hu, Haixia He, Wei Chen, Yu Sun

**Affiliations:** 1 Department of Otorhinolaryngology, The Central Hospital of Wuhan, Tongji Medical College, Huazhong University of Science and Technology, Wuhan 430014, China; 2 Department of Otorhinolaryngology, Union Hospital, Tongji Medical College, Huazhong University of Science and Technology, Wuhan 430022, China

**Keywords:** congential hearing loss, congential deafness, etiology, gene diagnosis

## Abstract

**Background:** Hearing loss (HL) is the most common disorder in newborns with a highly heterogeneous genetic background. Despite significant progress in screening and identifying genes related to congenital hearing loss, there are still candidate genes implicated in HL that remain undiscovered. **Methods:** We investigated HL in 43 Chinese families by segregating bilateral sensorineural HL via whole-exome sequencing (WES) and Sanger sequencing. **Results:** Variants were found in 10 known non-syndromic hearing loss (NSHL) genes, 5 known syndromic hearing loss (SHL) genes, and 1 candidate HL gene, *ATP7B*. RNA sequencing revealed *ATP7B* mRNA expression in developing and adult cochleae. The immunohistochemistry of the adult mouse cochlear tissue revealed the prominent expression of ATP7B in the organ of Corti and the spiral ganglion neuron. Overall, we propose a new candidate gene, *ATP7B*, for congenital hearing loss and novel variants in known HL genes, which expands our understanding of the etiology of HL. **Conclusions:** The next-generation sequencing could effectively improve the etiological diagnosis rate of congenital hearing loss in children.

## 1. Introduction

Hearing loss (HL) is the most common disorder and has an incidence of 2–3 out of 1000 live births [1]. Genetics account for at least 50% of congenital hearing loss [2]. Regional and ethnic differences in genetics between populations exist [3]. To date, more than 120 non-syndromic hearing loss (NSHL) genes have been identified [4,5]. *GJB2* is the most common gene associated with autosomal recessive (AR) NSHL among the East Asian population [6,7].

Identifying the genetic etiology of HL is essential for customizing personalized treatment plans, evaluating hearing recovery after cochlear implantation, and genetic counseling [8,9]. Patients carrying different pathogenic variants exhibit a wide spectrum of phenotypes and vastly differing outcomes after cochlear implantation; thus, a precise genetic diagnosis for HL patients may predict the potential benefits of cochlear implants [10]. Variants limited to pre-synaptic areas such as cochlear hair cells, supporting cells, or stria vascularis regions may result in better hearing recovery effects after cochlear implantation, while variants limited to the postsynaptic to the auditory central region may result in uneven or poor hearing recovery after cochlear implantation [10,11,12]. Genetic testing combined with genetic counseling for congenital hearing loss may also reduce the incidence of congenital hearing loss in newborns.

Despite significant progress in screening and identifying genes related to congenital hearing loss, there are still candidate genes implicated in HL that remain undiscovered [13,14,15,16]. Whole-exome sequencing (WES) has recently enhanced the discovery of rare or novel variants in known and candidate genes related to hearing loss. In this study, we investigated HL in 43 Chinese families by segregating bilateral sensorineural HL via WES and Sanger sequencing and identified rare and novel variants in known and candidate genes, which are valuable for a deeper understanding of the relationship between the genotype and phenotype of HL.

## 2. Materials and Methods

### 2.1. Sample Collection and Clinical Evaluation

Participants were enrolled from our outpatient department. Approval was granted by the Institutional Review Boards of Tongji Medical College, Huazhong University of Science and Technology (2022 S041). Before participation, the study was clearly explained to each participant or their parents. All participants or their parents signed written initial informed consent per the Declaration of Helsinki. All of the probands underwent evaluation, including family and clinical history; available medical records; physical examination; tympanometry; and audiological examinations, such as pure tone audiometry (PTA), audiometry Distortion Product Otoacoustic Emission (DPOAE), and auditory steady-state-evoked responses (ASSRs) or auditory brainstem responses (ABRs). Three to five milliliters of peripheral blood samples from the probands and related family members were collected and subjected to whole-exome sequencing. Further clinical evaluation was used to determine whether the patient and family members had the syndrome. Temporal bone magnetic resonance imaging (MRI) or computed tomography (CT) was used to identify any possible developmental malformation.

### 2.2. Whole-Exome Sequencing and Data Analyses

Genomic DNA (gDNA) samples from 43 probands from 43 families underwent WES. The method has been described in detail in our previous articles. gDNA was extracted from the samples using the QIAamp DNA Blood Maxi Kit(Qiagen Inc., Hilden, Germany). The fragmentation of the genomic DNA was performed to generate the exome library. The libraries were sequenced on the BGISEQ-500 platform(BGI Genomics, Wuhan, China). Sequencing data were compared with the human genome reference (GRCh37/hg19) to detect target regions, single-nucleotide variants (SNVs), and INDEL calling. Identified SNVs and indels were compared with the information available in multiple databases (National Center for Biotechnology Information GenBank database, the Database of Single Nucleotide Polymorphisms, and the 1000 Genomes Database). Pedigree mapping of the suspected variants was amplified via Sanger sequencing or WES.

### 2.3. RNA Expression Profiling of ATP7B in Mice Cochlear Tissues

Publicly available datasets in the Gene Expression Omnibus (GEO) database and SHIELD (Shared Harvard Inner-Ear Laboratory Database) were utilized for statistical analysis and the evaluation of *ATP7B* RNA expression levels in the mice cochlea. RNA sequencing data for hair cells and surrounding cells from the cochlea and utricle at different developmental stages (E16, P0, P4, and P7) originated from the GSE60019 dataset. We also analyzed the expression of the *ATP7B* genes in adult CBA/J mice cochlear RNA sequencing data from the GSE111347 dataset, which includes the transcriptome of the cochlear pillar and Deiters’ cells of adult CBA/J mice (28–35 days after birth), and the GSE56866 dataset, which also includes the transcriptome of cochlear inner hair cells (IHCs) and outer hair cells (OHCs) of either sex of adult CBA/J mice (25–35 days after birth). The cell-specific expression of the *ATP7B* gene in two datasets containing adult mouse cochlear RNA sequencing was comprehensively analyzed using Transcripts Per Kilobase Million (TPMs).

### 2.4. Immunofluorescence of ATP7B Expression in Cochlear Tissue

To observe the regions where *ATP7B* is expressed in the inner ears of auditory mature mice, we prepared cochlear samples and conducted immunofluorescence staining. Cochlear tissues were dissected from C57BL/6 mice on postnatal day 18 (P18). After the mice were euthanized, the cochlear tissues were carefully dissected from the temporal bones and rapidly moved into 4% paraformaldehyde at 4 °C overnight. We then treated the cochlear tissues with 10% EDTA-Na2 at 4 °C overnight to soften the tissues. The cochlear tissues were embedded in optimal cutting temperature compound (Servicebio, G6059) and sectioned on a microtome (Leica, CM1950, Wetzlar, Germany,) to 10 μm sections at −25 °C. The prepared tissue sections were stored at −80 °C. The cochlear tissue specimens were incubated in 10% donkey serum for 1 h at room temperature and then placed in ATP7B primary antibody (ABclonal, A5676, Wuhan, China, 1:200 diluted) and incubated overnight at 4 °C. Then, the cochlear tissue specimens were washed with 0.1% Tween20 in PBS four times and incubated with Alexa Fluor^®^488 Donkey anti-Rabbit secondary antibodies (AntGene, ANT024s, Wuhan, China) for 2 h at room temperature. After incubation with secondary antibodies, specimens were washed with 0.1% Tween20 in PBS three times. DAPI (AntGene, ANT165, Wuhan, China,) and phalloidin (Yeasen, 40736ES75, Shanghai, China) were used for nuclear and F-actin staining, respectively. Images were obtained with a Nikon Spatial Array Confocal (Nikon, Tokyo, Japan).

## 3. Results

### 3.1. The Pedigree Analysis of the Families in This Cohort

**Figure 1:**
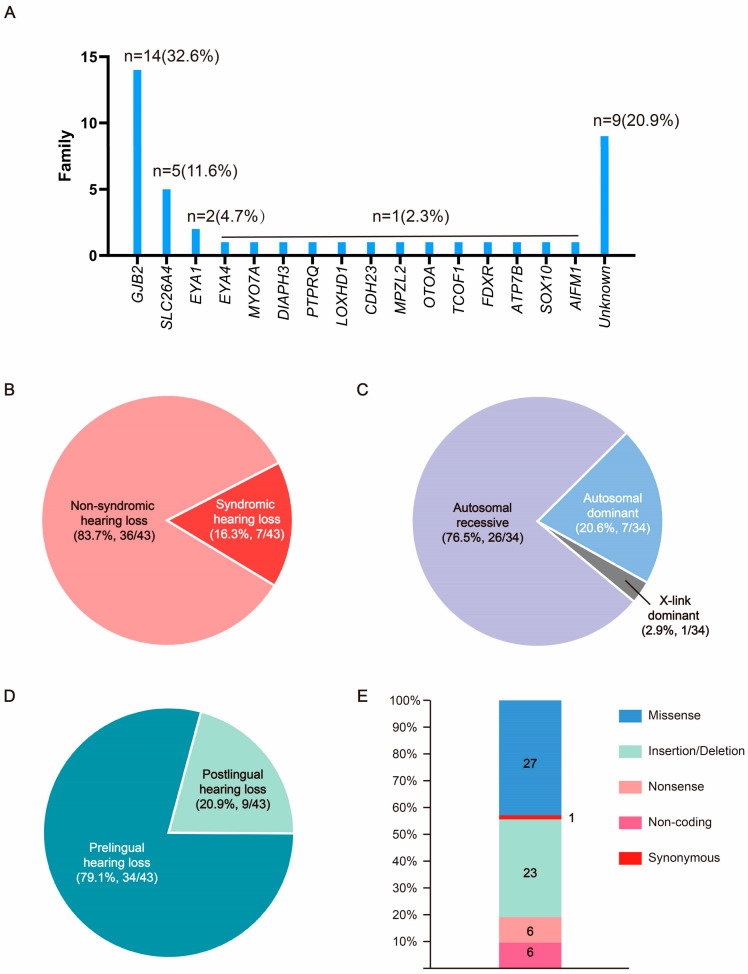
Analysis and overview of HL gene variants in this cohort. (**A**) Frequency of the known HI and candidate genes in Chinese families included in this cohort. (**B**) Proportions of non-syndromic hearing loss and syndromic hearing loss families in this cohort. (**C**) Proportions of hereditary mode of variants in known and novel candidate HL genes were identified. (**D**) Proportions of pre-lingual and post-lingual hearing loss of the probands in this cohort. (**E**) Proportions of variant types in known and novel candidate HL genes were identified.

This cohort consisted of 43 unrelated Chinese families. The participants in this study were recruited across the country, with the majority from Hubei province. A total of 79.1% (34/43) of families could be explained as carrying genomic variants, leaving 20.9% (9/43) of families without genetic causes identified through WES (Figure 1A). The number of NIHL families and SHL families was 36 (84.8%) and 7 (15.2%), respectively (Figure 1B). A total of 76.5% (26/34 genetic causes) of families exhibited a likely AR mode of inheritance. A total of 20.6% (7/34 genetic causes) of families showed a pattern of an autosomal dominant (AD) mode of inheritance. A total of 2.9% of families (1/34 genetic causes) had a possible X-linked dominant mode of inheritance (Figure 1C). A total of 79.1% (34/43) of families had members with pre-lingual HL (Figure 1D).

### 3.2. Bioinformatic and Molecular Analysis

Identified variants in this study include missense variants (27), insertion/deletion (23), nonsense (6), synonymous (1), and non-coding variants (6) (Figure 1E). Ten known NSHL genes were identified in 27 of the 43 families (Supplementary Table S1). These genes included *GJB2*, *SLC26A4*, *MYO7A*, *DIAPH3*, *PTPRQ*, *LOXHD1*, *CDH23*, *MPZL2*, *EYA4*, and *OTOA*. Five known SHL genes were identified in 6 of the 43 families (Supplementary Table S2). These genes included *TCOF1*, *EYA1*, *FDXR*, *AIFM1*, and *SOX10*. *ATP7B* was segregated in the SHL family as a candidate SHL gene (Supplementary Table S3). Variants in *GJB2* (14 families, 32.6%) and *SLC26A4* (5 families, 11.6%) accounted for the majority (44.2%). *EYA1* variants were found in two families, and variants in 13 other genes were only observed in one family. Twenty of the variants were reported to be associated with the occurrence of hearing loss, while thirteen of the novel variants were found to segregate in 11/43 HL families, inlcuding *DIAPH3* c.2256_2257insT, *PTPRQ* c.6293T>C, *CDH23* c.4859T>A, *MPZL2* c.393_436+21del, *TCOF1* c.3997_4007del, *LOXHD1* c.2438T>A and c.1759C>T, *EYA1* c.1350_1353delTAATinsCAGACA, *FDXR* c.1069G>T and c.364C>T, ATP7B c.4014T>A, *SOX10* c.133del, and *AIFM1* c.1771-14T>A.

#### 3.2.1. Non-Syndromic Hearing Loss Gene Variants

**Figure 2:**
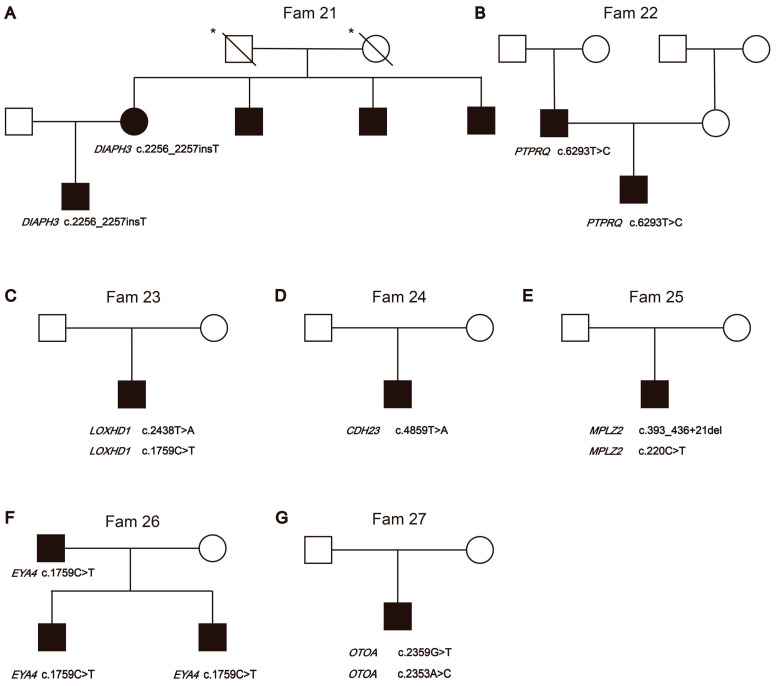
Pedigrees of families’ segregation of novel non-syndromic hearing loss gene variants. The segregation of non-syndromic hearing loss genes is shown in the respective families. (**A**) Fam21, (**B**) Fam22, (**C**) Fam23, (**D**) Fam24, (**E**) Fam25, (**F**) Fam26, and (**G**) Fam27. * indicates individuals whose audiological examination results cannot be obtained.

Fourteen families segregated variants in *GJB2* (OMIM: 121011), while five families segregated variants in *SLC26A4* (OMIM: 605646), which are the most common HL genes in East Asia [17,18]. All the variants (N = 12) identified in these families were classified as pathogenic and likely pathogenic based on the ACMG-AMP classification guidelines for HL. *MYO7A* (OMIM: 276903) variants c.1183C>T (p.Arg395Cys) and c.3696_3706del (p.Arg1232SerTer72) were segregated from the NSHL phenotype of the proband in Fam 20. Fam 21 was found to have a *DIAPH3* (OMIM: 603550) c.2256_2257insT (p.Ser752SerfsTer12) variant segregating with the HL phenotype. In Fam 21, the mother of the proband was confirmed by Sanger sequencing to carry *DIAPH3* c.2256_2257insT (Figure 2A). The mother’s three brothers also affected HL, although they did not undergo genetic testing. *PTPRQ* (OMIM: 603317) is associated with ADHL (OMIM: 617663) and ARNSHL (OMIM: 613391). Novel heterozygous variants *PTPRQ* c.6293T>C (p.Leu2098Ser) were segregated in Fam 22 (Figure 2B). The proband showed normal vestibular function until now. *LOXHD1* (OMIM: 613072) is associated with ARNSHL (OMIM: 613079). Novel compound heterozygous variants *LOXHD1* c.2438T>A (p.Leu813Ter) and c.1759C>T (p.Arg587Trp) were segregated in Fam 23 (Figure 2C). *CDH23* (OMIM: 605516) was involved in Usher syndrome 1D (OMIM: 601067) and ARNSHL (OMIM: 601386). Fam 24 was found to have Homozygous *CDH23* c.4859T>A (p.Val1620Glu) segregating with the profound HL phenotype (Figure 2D). The proband was 13 years old at the time of testing and had not yet exhibited vestibular dysfunction or pigmentary retinopathy. Fam 25 segregates compound heterozygous variants *MPZL2* (OMIM: 604873) c.393_436+21del and c.220C>T (p.Gln74Ter) (Figure 2E). *MPZL2* was associated with ARNSHL (OMIM: 618145). The features of Fam26 are an HL father and two HL boys, which both carry the *EYA4* (OMIM: 603550) c.1759C>T (p.Arg587Ter) variant (Figure 2F). *EYA4* was associated with ARNSHL (OMIM: 601316). No clinical signs were observed after the genetics tests that would indicate that affected family members have disorders affecting other organs related to syndromic HL. We also identified novel compound heterozygous variants c.2359G>T (p.Glu787Ter) and c.2353A>C (p.Thr785Pro) in *OTOA* (Figure 2G) in Fam 27. *OTOA* (OMIM: 607038) was associated with ARNSHL (607039). The proband in Fam27 has not yet shown any phenotype other than HL. The hearing thresholds and the age of HL onset of all patients were recorded (Supplementary Table S4).

#### 3.2.2. Syndromic Hearing Loss Gene Variants

**Figure 3:**
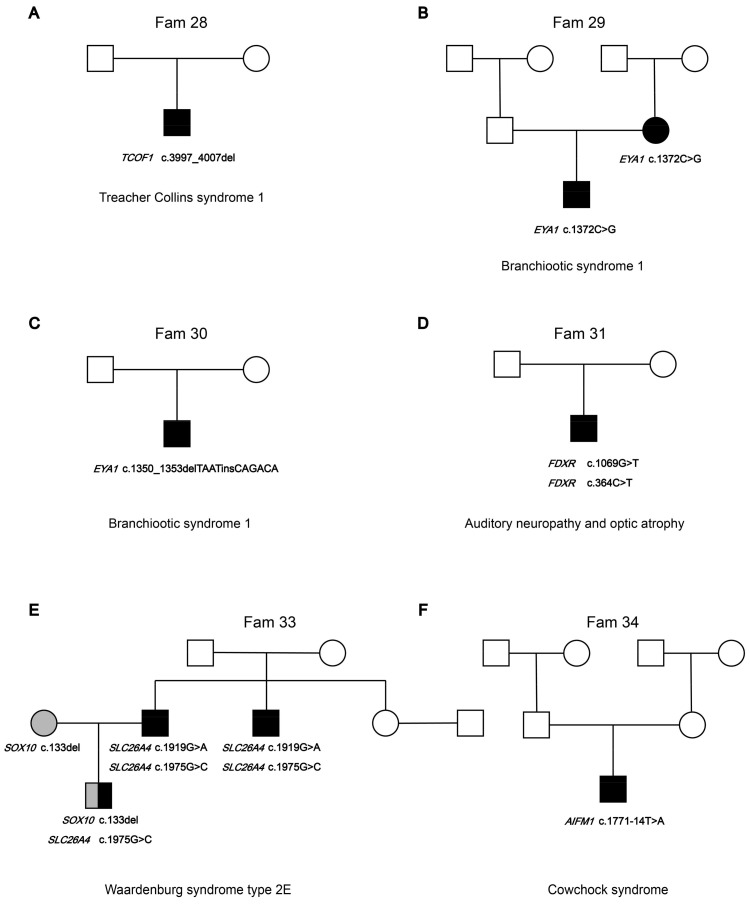
Pedigrees of families’ segregation of syndromic hearing loss gene variants. The segregation of syndromic hearing loss genes is shown in the respective families. (**A**) Fam28, (**B**) Fam29, (**C**) Fam30, (**D**) Fam31, (**E**) Fam33, and (**F**) Fam34.

Novel heterozygous variants *TCOF1* (OMIM: 606847) and c.3997_4007del (p.Ser1333GlnfsTer16) were identified to be likely causes of SHL in Fam 28 (Figure 3A). The proband in Fam28 was clinically diagnosed with Treacher Collins syndrome (OMIM:154500). Two families (Fam29 and Fam30) segregate variants *EYA1* (OMIM: 601653), c.1225C>T (p.Asn451ArgfsTer18), and c.1350_1353delTAATinsCAGACA (p.Pro458Ala) (Figure 3B,C). *EYA1* is associated with Branchiootic syndrome 1 (OMIM: 602588). Both probands were observed to have clinical features of branchial cervical fistulae, preauricular pits, and cup-shaped Pinnae. Novel compound heterozygous variants *FDXR* c.1069G>T (p.Val357Leu) and c.364C>T (p.Arg122Cys) were identified to be likely causes of SHL in Fam 31 (Figure 3D). *FDXR* (OMIM: 103270) variants *FDXR* c.364C>T (p.Arg122Cys) and c.1069G>T (p.Val357Leu) could cause auditory neuropathy and optic atrophy (OMIM: 617717) or multiple mitochondrial dysfunction syndrome 9B (OMIM: 620887). Probands with severe HL in Fam 31 have bilateral optic nerve atrophy and retinitis pigmentosa, while the brother of the probands in Fam 31 has Down syndrome (OMIM: 190685). Sanger sequencing confirms that the mother of the proband carries a heterozygous variant, c.364C>T (p.Arg122Cys), in *FDXR*, and the father of the proband does not carry an *FDXR* variant. In Fam 33, novel heterozygous variants *SOX10* (OMIM: 602229) and C.133del (p.Gly38AlafsTer71) and heterozygous variants *SLC26A4* (OMIM: 605646) and c.1975G>C were both identified in the proband (Figure 3E). *SOX10* variants have been associated with Waardenburg syndrome 2E (OMIM:611584) and Waardenburg syndrome 4C (OMIM:613266). The proband has blue eyes and profound HL. The mother of the proband in Fam 33 also has blue eyes and HL and was identified as carrying heterozygous variant C.133del in *SOX10* via WES sequencing. The father of the proband in Fam 33, with the HL phenotype, was identified as carrying compound heterozygous variants c.1975G>C and c.1919G>A (p.Trp640Ter) in *SLC26A4*. In addition, the younger brother of the proband’s father in Fam 33 also suffered from HL and was identified to carry a compound heterozygous variant c.1975G>C and c.1919G>A in *SLC26A4*. Additionally, the proband in Fam 34 was identified to carry novel variant *AIFM1* c.1771-14T>A (Figure 3F). *AIFM1* (OMIM: 300169) has been associated with X-linked deafness-5 with peripheral neuropathy (DFNX5, OMIM: 300614), Cowchock syndrome (OMIM: 300169), and combined oxidative phosphorylation deficiency 6 (OMIM: 300816). The proband in Fam 34 showed delayed development at 6 months after birth. Then, the proband underwent a series of clinical examinations and was diagnosed with profound sensorineural hearing loss. An MRI showed ventricular enlargement. The proband had multiple epileptic seizures. By age 5 years, the proband could not stand or walk alone, had poor eye contact, and was almost unresponsive to external sound stimuli. Recently, the proband underwent cochlear implantation at the age of 6 and received continuous speech and motor rehabilitation training. However, the parents of the proband do not believe that cochlear implantation and speech rehabilitation training increase the proband’s response to external sound stimuli. The parents of the proband do not carry the variant in *AIFM1*. The *AIFM1* variant in the proband may be a spontaneous mutation.

#### 3.2.3. Hearing Loss Candidate Gene Variants

**Figure 4:**
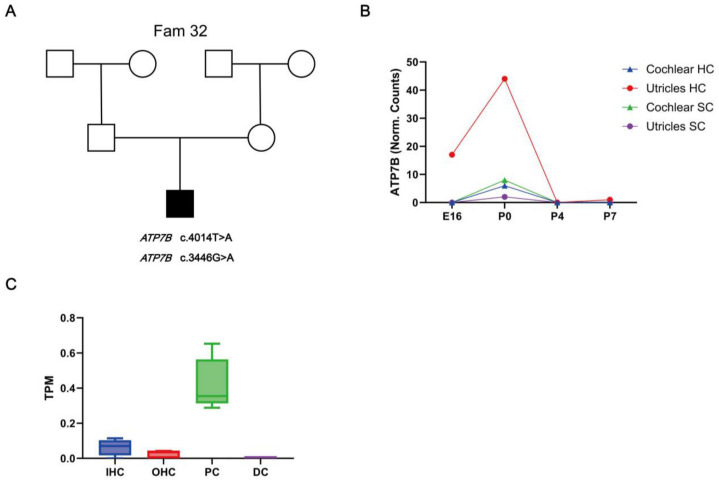
Pedigrees of Fam32 segregation *ATP7B* variants and expression of novel candidate gene *ATP7B* in the development of adult cochlear. (**A**) Pedigrees of Fam32 segregation *ATP7B* variants. (**B**) *ATP7B* mRNA expression in the hair cells (HCs) and surrounding cells (SCs) of mouse cochlea and utricle during four different developmental stages: E16, P0, P4, and P7. RNA sequencing data of hair cells (GFP+) and surrounding cells (GFP−) from the cochlear and utricles of mice expressing EGFP under the Pou4f3 promoter during developmental stages. (**C**) *ATP7B* mRNA expression in the inner ear cells of adult CBA/J mice. *ATP7B* mRNA expression in Deiters’ cells (DCs), pillar cells (PCs), inner hair cells (IHCs), and outer hair cells (OHCs) were evaluated. The y-axis represents the gene expression normalized to transcripts per million (TPM). Data for (**A**,**B**) were obtained from Gene Expression Omnibus (GEO) database and SHIELD (Shared Harvard Inner-Ear Laboratory Database).

ATP7B (OMIM: 606882) is associated with autosomal recessive disorder Wilson disease (OMIM: 277900). In Fam 32, the proband was identified to carry a compound heterozygous variant of ATP7B c.4014T>A (p.Ile1338Ile) and c.3446G>A (p.Gly1149Glu) (Figure 4A). The heterozygous variant of *ATP7B* c.4014T>A was also identified in the father of the proband, while the heterozygous variant of *ATP7B* c.3446G>A was identified in the mother of the proband. Thus, *ATP7B* compound heterozygous c.4014T>A and c.3446G>A may show an AR mode of inheritance.

### 3.3. Expression of HL Candidate Genes ATP7B in the Mouse Inner Ear

We used various publicly available RNA sequencing and microarray datasets to investigate the expression of HL candidate gene *ATP7B* in the developing and adult mouse cochlear. *ATP7B* shows low mRNA expression in the developing cochlear epithelium, including cochlear hair cells (HCs) and cochlear surrounding cells (SCs) throughout E14, P0, P4, and P7 (Figure 4B). The expressions of *ATP7B* mRNA were detected in pillar cells (PC) in adult CBA/J mice cochlear, while less *ATP7B* mRNA was detected in inner hair cells (IHCs), outer hair cells (OHCs), and Deiters’ cells (DCs) (Figure 4C).

**Figure 5:**
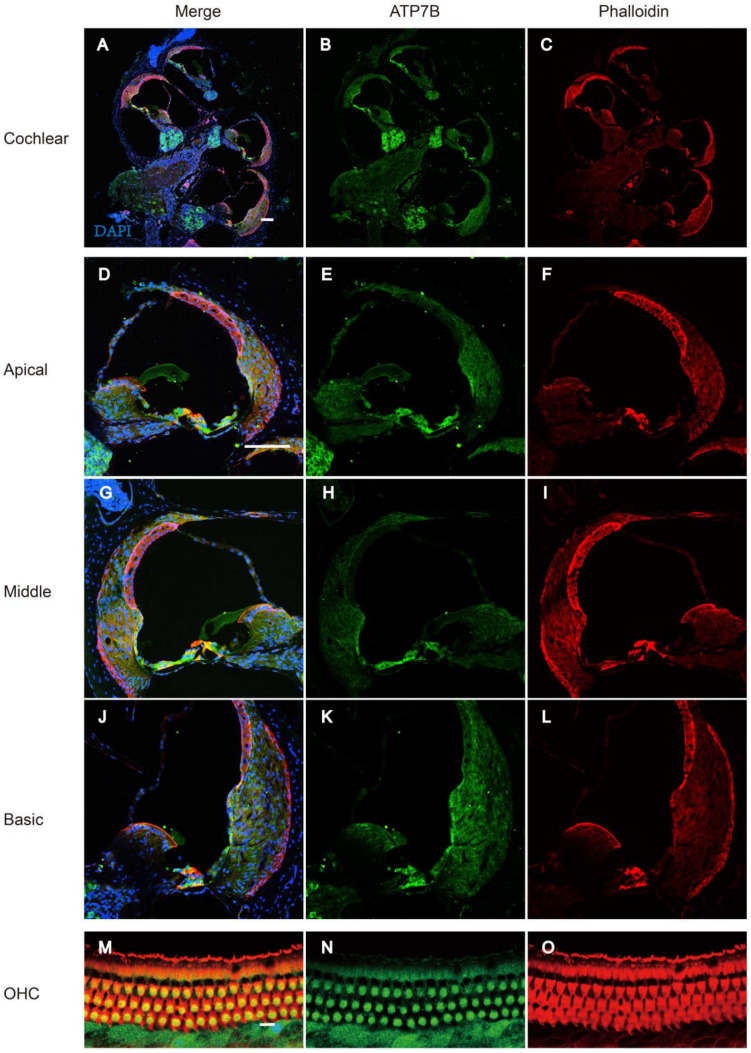
Immunostaining of ATP7B in the cochlear of C57BL/6 mice at P18. (**A**–**C**) Whole-mount immunostaining of the cochlear of P18 C57BL/6 mice. Immunostaining of ATP7B (Green) is observed in both spiral ganglion cells and organ of Corti. Scale bar: 100 μm. (**D**–**L**) Immunostaining of ATP7B (green) in regions of the OC at the apical, medial, and basal turns of the cochlea specimens of C57BL/6 mice at P18. Immunostaining of ATP7B (green) is observed in IHCs, OHCs, DCs, PCs, and Claudius cells. Scale bar: 100 μm. (**M**–**O**) Immunostaining of ATP7B (green) in cytoplasmic region of the cochlear outer hair cells of C57BL/6 mice at P18. Nucleus was stained with DAPI (blue). F-actin was stained with phalloidin (red). Scale bar: 10 μm.

**Figure 6:**
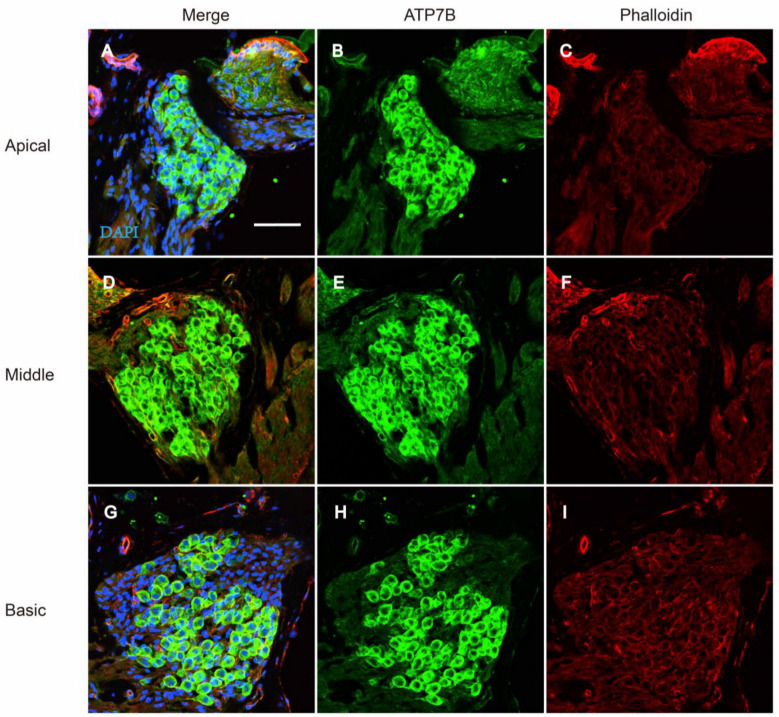
Immunostaining of ATP7B in the spiral ganglion cell of C57BL/6 mice at P18. (**A**–**I**) Whole-mount immunostaining of ATP7B in the spiral ganglion cell of P18 C57BL/6 mice. Nucleus was stained with DAPI (blue). F-actin was stained with phalloidin (red). Immunostaining of ATP7B (green) is observed in spiral ganglion cells at apical, middle, and basic turns. Scale bar: 50 μm.

The onset of mice hearing around P12 and the hearing threshold of mice remains stable around P18. To detect the expression of ATP7B protein in the adult mouse cochlea, we performed immunofluorescence staining on cochlear tissues of C57BL/6 mice at P25. ATP7B was detected as mainly expressed in the spiral ganglions (SGNs) and organ of Corti (OC), including IHC, OHC, PC, DC, and Claudius’ cells (CCs) (Figure 5A–L). Immunofluorescence results also demonstrate that ATP7B was expressed in the cytoplasmic region of the cochlear outer hair cells (Figure 5M–O) and spiral ganglion cells (Figure 6A–I).

## 4. Discussion

In this study, we investigated HL in 44 China families that segregate bilateral sensorineural HL. In total, we identified variants in 15 known HL genes. Genetic causes account for at least 79.1% of the studied families, which is higher than an earlier result [19]. Via the present data, we re-evaluated the contribution of GJB2 as 32.6% (14/43) among all families investigated, while the contribution of SLC26A4 was determined to be 11.6% (5/43), which suggests the prioritization of GJB2 and SLC26A4 in the clinical practice of NIHL. This is consistent with the results in the previous Chinese HL population cohort [20,21,22]. The main pathogenic variants in GJB2 were c.235delC, c.109G>A, c.299_300delAT, and c.176_191del, with allele frequencies of 11.63%, 10.47%, 3.49%, and 3.49%, respectively. The main pathogenic variants in SLC26A4 were c.919-2A>G and c.2168A>G, with allele frequencies of 2.33% and 1.16%, respectively.

The molecular mutation spectrum of the hereditary HL gene and its epidemic characteristics exhibit distinct racial and geographic area specificity [6]. Dai et al. reported the results of screening the 235delC mutation in NSHL patients from 26 different regions of China and showed that the allele frequency of c.235delC varied with regions from 0.9% to 19.5%, yielding a nationwide average frequency of 12.0% in China based on the populations studied [7]. The allele frequency of c.235delC of Man, Mon, Han, and Hui minorities in Xinjiang and minorities in Southwestern China and Tibet is 23.7%, 18.3%, 13.0%, 10.1%, 5.1%, 4.1%, and 0.8%, respectively [7]. In another cohort conducted in northwest regions of China, the frequency of pathogenic mutations for GJB2 was 8.27%, and the pathogenic allele frequency for GJB2 was 11.73%. GJB2 c.235delC and c.35delG had the highest allele frequencies, at 5.45% and 2.29% [23]. The allele frequency of GJB2 c.235delC in HL patients of Hui (7.49%), Dongxiang (3.79%), Tibetan (2.34%), and Uygur (5.23%) nationality was significantly lower than that in patients of Han ethnicity (10.34%). The differences in allelic frequencies for GJB2 between Hubei Province and the northwest region of China may be related to the racial differences among the participants enrolled in the different cohorts. However, the allele frequency of GJB2 c.235delC in HL patients of Han nationality in northwest regions of China (10.34%) was similar to our statistical results in Hubei Province (11.63%). Li et al. reported that the allelic frequencies of GJB2 235delC in patients of Uigur nationality and Han nationality HL were 5.7% and 9.8%, and the allelic frequencies of 299–300delAT were 0.8% and 5.5% [24]. The allele frequencies of c.235delC are lower frequency in the minorities in the southwest and the northwest regions than in the majorities in Han. These studies suggest the need to generate gene diagnosis strategies based on the prevalence of HL genes in regions and ethnic groups. Other studies have also reported the allele frequencies of GJB2 and SLC26A4 variants in HL patients from different regions of China. The allele frequency of GJB2 c.235delC, c.109G>A, c.299_300delAT, and c.176-191del16 ranged from 1.44% to 15.62%, 3.26% to 16.84%, 0.15% to 2.06%, and 0.17% to 0.6%, respectively. The allele frequency of SLC26A4 c.919-2A>G and c.2168A>G ranged from 3.66% to 12.46% and 0.11% to 1.7%, respectively. The differences between different studies may be related to factors such as region, genetic testing methods, and the number of subjects enrolled. In addition, c.235delC is considered to be the most common GJB2 mutation in East Asian populations. However, the allele frequency of c.235delC varies among China (12.0%), South Korea (9.3%), and Japan (3.9%). The allele frequency of GJB2 c.235delC seems to decline from China to South Korea to Japan [25,26,27]. It should be noted the differences in the frequency of the c.235del allele in different countries may be related to the number of studies included in the analysis and the number of HL patients [7].

Early genetic testing for deafness has potential benefits for patients. It has been reported that GJB2 mutations may exhibit a phenotype–genotype correlation [27,28,29]. Thus, it is important to conduct genetic testing on HL patients and analyze the frequency distribution and genotype—phenotype correlation of GJB2 mutations in hearing loss patients to support genetic diagnosis and counseling. In addition, Wu et al. reported that patients with GJB2 or SLC26A4 mutations who received cochlear implants before 3.5 years of age showed significantly higher CAP/SIR scores than those without mutations at post-implant year 3 [30]. Patients with early cochlear implants with GJB2 or SLC26A4 mutations demonstrated better hearing than those without mutations, which may be attributed to the pathogenic consequences of GJB2 and SLC26A4 variants being confined to the cochlea while the neural integrity of the auditory system was reserved. In this study, 83.7% (36/43) of families were diagnosed with NSHL. Childhood HL is one of the most heterogeneous diseases. Thus, it should be noted that patients with hereditary hearing loss may only have isolated hearing loss phenotypes in childhood, which are often misdiagnosed as non-syndromic hearing loss in clinical practice, ignoring other systemic diseases that may occur after puberty or adulthood [31,32,33]. Genetic diagnosis helps detect additional phenotypes earlier or alert patients to potential risks. MYO7A variants may cause either NSHI or Usher syndrome (USH). Liu et al. reported that an NSHL proband carried MYO7A heterozygous variants of c.1183C>T and c.1496T>C [34]. Xia et al. reported the co-segregation of the homozygous MYO7A c.3696_3706del variant with the phenotype of deafness and progressive visual loss in the USH family [35]. Until now, the proband in Fam 20 carried MYO7A heterozygous variants of c.1183C>T, and c.3696_3706del has not shown any ophthalmic or vestibular phenotype. Similarly, PTPRQ variants could cause autosomal recessive or autosomal dominant congenital sensorineural hearing loss, with or without vestibular dysfunction in infancy or early childhood [36]. It cannot be ruled out that patients currently diagnosed with NSHL may develop late-onset syndrome symptoms in the future [37,38]. We will closely follow up with the proband in order to intervene in time. Accurate genetic diagnosis could also help us develop targeted and efficient strategies for SHL patients [39,40,41,42]. In this study, SHL patients were found in seven families (16.2%). It has been reported that prolonged treatment with riboflavin may result in some mild improvement in the ataxia caused by AIFM1 mutation. Ghezzi et al. reported that riboflavin supplementation could partially correct increased cell death and severe respiratory chain deficiency [41,42]. After the proband was identified as carrying the AIFM1 variant, riboflavin therapy was recommended. So far, no significant improvement in the patient’s symptoms has been observed. Genetic testing needs to be combined with clinical follow-up.

In this study, we identified novel compound heterozygous variants c.4014T>A and c.3446G>A in the candidate gene ATP7B. Variant ATP7B c.3446G>A was predicted to be likely pathogenic [43], while the clinical features of the ATP7B c.4014T>A carrier could have conflicting interpretations according to gnomAD. It has been reported that synonymous mutation c.4014T>A (p.Ile1338Ile) could cause exon skipping in the ATP7B mRNA transcript [44]. ATP7B encodes a copper transporter. Variants in ATP7B resulted in a rare autosomal recessive disorder of copper metabolism, Wilson disease (WD), also called progressive lenticular degeneration [43,45,46]. The age range of onset of WD is wide, and symptoms of WD can appear at any age [47,48]. ATP7B shows low mRNA expression in the developing cochlear epithelium, while in adult mice, ATP7B protein is expressed in OC and SGN cells. Mutations in ATP7B may lead to the accumulation of copper in organs, which can cause damage and death to hair cells and spiral ganglion neurons. These findings suggest that ATP7B may function in mature cochlear cells and may be associated with delayed progressive hearing loss. Long-term hearing tests need to be conducted to clarify whether the proband has progressive hearing loss. The ATP7B deficiency animal and in vitro models may also provide assistance in determining the phenotype and mechanism of hearing loss and cell damage. More experiments in vivo and vitro need to be conducted to develop and validate new hearing protection drugs. In recent years, research on next-generation gene sequencing has reported many HL candidate genes across different regions and ethnic groups. Our understanding of the relationship between the HL genetic etiology and phenotype–genotype correlation is rapidly increasing. Identifying deaf variants and providing genetic counseling could indeed reduce the risk of HL in the next generation of newborns. However, there is still a lot of work to be completed.

## 5. Conclusions

In this study, we used WES for the genetic diagnosis of 43 bilateral sensorineural HL probands. A total of 13 novel variants were identified in 10 HL genes and a novel candidate gene, which enlarged the variant spectrum of HL genes in patients of Han nationality. We hope this study may help inform the genetic diagnosis and prenatal genetic diagnosis of HL couples and add to the theoretical basis for deafness diagnosis, accurate genetic counseling, and procreation guidance.

## Data Availability

The original contributions presented in this study are included in the article/Supplementary Materials. Further inquiries can be directed to the corresponding authors.

## Supplementary Materials

The following supporting information can be downloaded at: https://www.mdpi.com/article/10.3390/biomedicines12122657/s1, Table S1: Variants identified in the non-syndromic hearing loss families; Table S2: Variants identified in the syndromic hearing loss families; Table S3: Hearing loss candidate variants segregated in Fam32; Table S4: Hearing threshold of the probands.
