# Supplementary material for: Next-Generation Sequencing of Chinese Children with Congenital Hearing Loss Reveals Rare and Novel Variants in Known and Candidate Genes
Source: Biomedicines. 2024 Nov 21;12(12):2657. doi: 10.3390/biomedicines12122657 (PMC11673014; doi:10.3390/biomedicines12122657)
Supplement: Supplementary file 1 [file biomedicines-12-02657-s001.zip › biomedicines-3301220-supplementary.pdf]

Table S1. Variants identified in the non-syndromic hearing loss families.

Family	Gene	Nucleotide change	Protein change	Inh	GT	Mutation type
Fam1	*GJB2*	c.235del	p.Leu79CysfsTer3	AR	Hom	Deletion
Fam2	*GJB2*	c.99del	p.Met34Ter	AR	Het	Deletion
		c.299_300del	p.His100ArgfsTer14	AR	Het	Deletion
Fam3	*GJB2*	c.235del	p.Leu79CysfsTer3	AR	Hom	Deletion
						Deletion
Fam4	*GJB2*	c.109G>A	p.Val37Ile	AR	Hom	Missense
Fam5	*GJB2*	c.139G>T	p.Glu47Ter	AR	Het	Nonsense
		c.176_191del	p.Gly59AlafsTer18	AR	Het	Deletion
Fam6	*GJB2*	c.109G>A	p.Val37Ile	AR	Hom	Missense
Fam7	*GJB2*	c.235del	p.Leu79CysfsTer3	AR	Hom	Deletion
Fam8	*GJB2*	c.235del	p.Leu79CysfsTer3	AR	Het	Deletion
		c.176_191del	p.Gly59AlafsTer18	AR	Het	Deletion
Fam9	*GJB2*	c.109G>A	p.Val37Ile	AR	Hom	Missense
Fam10	*GJB2*	c.109G>A	p.Val37Ile	AR	Hom	Missense
Fam11	*GJB2*	c.235del	p.Leu79CysfsTer3	AR	Het	Deletion
		c.299_300del	p.His100ArgfsTer14	AR	Het	Deletion
Fam12	*GJB2*	c.235del	p.Leu79CysfsTer3	AR	Hom	Deletion
						Deletion
Fam13	*GJB2*	c.257C>G	p.Thr86Arg	AR	Het	Missense
		c.109G>A	p.Val37Ile	AR	Het	Missense
Fam14	*GJB2*	c.299_300delAT	p.His100ArgfsTer14	AR	Het	Deletion
		c.176_191del	p.Gly59AlafsTer18	AR	Het	Deletion
Fam15	*SLC26A4*	c.919-2A>G	-	AR	Het	Noncoding
		c.281C>T	p.Thr94Ile	AR	Het	Missense
Fam16	*SLC26A4*	c.1594A>C	p.Ser532Arg	AR	Het	Missense
		c.2168A>G	p.His723Arg	AR	Het	Missense
Fam17	*SLC26A4*	c.919-2A>G	-	AR	Hom	Noncoding
Fam18	*SLC26A4*	c.2009T>C	p.Val670Ala	AR	Hom	Missense
Fam19	*SLC26A4*	c.919-2A>G	-	AR	Het	Noncoding
		c.2168A>G	p.His723Arg	AR	Het	Missense
Fam20	*MYO7A*	c.1183C>T	p.Arg395Cys	AR	Het	Missense

Family	Gene	Nucleotide change	Protein change	Inh	GT	Mutation type
Fam21	*DIAPH3*	c.3696_3706del	p.Arg1232SerTer72	AR	Het	Deletion
		c.2256_2257insT	p.Ser752SerfsTer12	AD	Het	Deletion Insertion
Fam22	*PTPRQ*	c.6293T>C	p.Leu2098Ser	AD	Het	Missense
Fam23	*LOXHD1*	c.2438T>A	p.Leu813Ter	AR	Het	Nonsense
		c.1759C>T	p.Arg587Trp	AR	Het	Missense
Fam24	*CDH23*	c.4859T>A	p.Val1620Glu	AR	Hom	Missense
Fam25	*MPZL2*	c.220C>T	p.Gln74Ter	AR	Het	Nonsense
		c.393_436+21del	-	AR	Het	Noncoding
Fam26	*EYA4*	c.1759C>T	p.Arg587Ter	AD	Het	Nonsense
Fam27	*OTOA*	c.2359G>T	p.Glu787Ter	AR	Het	Nonsense
		c.2353A>C	p.Thr785Pro	AR	Het	Missense

[1] Inh mode of inheritance, hom homozygote, het heterozygote, GT patient genotype.

Table S2. Variants identified in the syndromic hearing loss families.

Family	Gene	Nucleotide change	Protein change	Inh	GT	Mutation type
Fam28	*TCOF1*	c.3997_4007del	p.Ser1333GlnfsTer16	AD	Het	Deletion
Fam29	*EYA1*	c.1372C>G	p.Pro458Ala	AD	Het	Missense
Fam30	*EYA1*	c.1350_1353delTAAT insCAGACA	p.Asn451ArgfsTer18	AD	Het	Deletion/ Insertion
Fam31	*FDXR*	c.1069G>T	p.Val357Leu	AR	Het	Missense
	FDXR	c.364C>T	p.Arg122Cys	AR	Het	Missense
Fam33	*SOX10*	C.133del	p.Gly38AlafsTer71	AD	Het	Deletion
	SLC26A4	c.1975G>C	p.Val659Leu	AR	Het	Missense
	SLC26A4	c.1919G>A	p.Trp640Ter	AR	Het	Nonsense
Fam34	*AIFM1*	c.1771-14T>A	-	X-link	Het	Noncoding

[2] Inh mode of inheritance, hom homozygote, het heterozygote, GT patient genotype.

Table S3. Hearing loss candidate variants segregated in Fam32.

Family	Gene	Nucleotide change	Protein change	Inh	GT	Mutation type
Fam32	*ATP7B*	c.4014T>A	p.Ile1338Ile	AR	Het	Synonymous
	ATP7B	c.3446G>A	p.Gly1149Glu	AR	Het	Missense

[3] Inh mode of inheritance, hom homozygote, het heterozygote, GT patient genotype.

Table S4. Hearing threshold of the probands

**Family**	**Gender**	**Age**	**Age at HL onset**	**Left ear Hearing threshold (dBnHL)**	**Right ear Hearing threshold (dBnHL)**
Fam1	Female	13 years	5 years	80	80
Fam2	Female	4 years	2 years	70	60
Fam3	Female	5 years	at birth	85	85
Fam4	Female	11 month	at birth	40	40
Fam5	Male	11 month	at birth	50	50
Fam6	Male	8 years	2 years	50	50
Fam7	Male	4 years	at birth	85	80
Fam8	Male	4 years	at birth	45	45
Fam9	Female	7 month	at birth	65	65
Fam10	Female	1 years	1 years	45	35
Fam11	Male	6 years	1 years	90	85
Fam12	Female	19 years	at birth	90	81
Fam13	Female	5 month	at birth	75	81
Fam14	Female	5 month	at birth	70	66
Fam15	Male	7 years	at birth	100	100
Fam16	Female	10 years	5 years	95	95
Fam17	Male	8 years	at birth	105	105
Fam18	Female	14 years	at birth	110	110
Fam19	Female	3 years	1 years	95	95
Fam20	Male	12 years	at birth	90	100
Fam21	Male	7 years	7 years	45	40
Fam22	Female	8 years	at birth	65	70
Fam23	Female	10 years	4 years	90	90
Fam24	Female	13 years	at birth	100	100
Fam25	Male	34 years	at birth	50	50
Fam26	Male	24 years	at birth	60	53
Fam27	Male	15 years	at birth	100	100
Fam28	Female	15 years	at birth	55	60
Fam29	Male	21 years	at birth	45	50
Fam30	Female	11 years	at birth	60	60
Fam31	Female	14 years	6 years	48	70
Fam32	Male	18 years	12 years	55	50
Fam33	Male	17 years	at birth	90	90
Fam34	Male	6 years	at birth	97	97
Fam35	Male	6 years	at birth	65	65
Fam36	Female	6 years	at birth	65	65

Fam37	Male	5 years	at birth	75	75
Fam37	Female	2 years	at birth	75	75
Fam38	Female	9 years	at birth	80	80
Fam39	Female	5 years	at birth	105	105
Fam40	Male	9 years	at birth	90	90
Fam41	Male	5 years	at birth	95	35
Fam42	Male	7 years	at birth	105	105
Fam43	Female	20 years	at birth	47	42
